# A Novel Multi-Feature Fusion Method in Merging Information of Heterogenous-View Data for Oil Painting Image Feature Extraction and Recognition

**DOI:** 10.3389/fnbot.2021.709043

**Published:** 2021-07-12

**Authors:** Tong Chen, Juan Yang

**Affiliations:** ^1^Basic College, Shanghai Institute of Visual Arts, Shanghai, China; ^2^Department of Design Art, Huaiyin Institute of Technology, Huaiyin, China

**Keywords:** heterogenous-view data, multi-feature fusion, support vector machine, K-nearest neighbor, oil painting image feature extraction, faster RCNN

## Abstract

The art of oil painting reflects on society in the form of vision, while technology constantly explores and provides powerful possibilities to transform the society, which also includes the revolution in the way of art creation and even the way of thinking. The progress of science and technology often provides great changes for the creation of art, and also often changes people's way of appreciation and ideas. The oil painting image feature extraction and recognition is an important field in computer vision, which is widely used in video surveillance, human-computer interaction, sign language recognition and medical, health care. In the past few decades, feature extraction and recognition have focused on the multi-feature fusion method. However, the captured oil painting image is sensitive to light changes and background noise, which limits the robustness of feature extraction and recognition. Oil painting feature extraction is the basis of feature classification. Feature classification based on a single feature is easily affected by the inaccurate detection accuracy of the object area, object angle, scale change, noise interference and other factors, resulting in the reduction of classification accuracy. Therefore, we propose a novel multi-feature fusion method in merging information of heterogenous-view data for oil painting image feature extraction and recognition in this paper. It fuses the width-to-height ratio feature, rotation invariant uniform local binary mode feature and SIFT feature. Meanwhile, we adopt a modified faster RCNN to extract the semantic feature of oil painting. Then the feature is classified based on the support vector machine and K-nearest neighbor method. The experiment results show that the feature extraction method based on multi-feature fusion can significantly improve the average classification accuracy of oil painting and have high recognition efficiency.

## Introduction

Feature extraction is the basis of object classification. Early studies tend to use shape features to describe the object, such as area, aspect ratio, duty ratio, etc,. With the development of object classification, more and more researches focus on the object features extraction and the construction of strong classifiers.

Bharath and Dhivya (2014) proposed a method of tracking and classifying objects based on features such as speed. Mithun et al. (2012) proposed a vehicle detection and classification method based on spatial and temporal features to solve the occlusion problem in traffic videos and the small difference between vehicles and the background. This method used the shape, texture and other features of moving object segmentation area, and adopted K-Nearest Neighbor (KNN) method to classify the object, which obtained better effect. Tian et al. (2019) classified the objects by combining periodicity with geometric shape features, but this method had high computational complexity. Dai et al. (2019) extracted four shape features of the object image area, took the obtained object description feature vector as the input of BP neural network. So a pedestrian and bicycle recognition method based on BP neural network was proposed. However, this method only classified several kinds of objects with different shape features. The scalable of this method was poor. Wang et al. (2020) extracted object features such as area, tightness, speed and length-width ratio of an external rectangular box. Then these features were trained and classified to achieve the purpose of foreground object recognition. On the basis of object area detection, Wu et al. (2013) took the centroid of the object area as the center, used multiple corner points detected in the object area to construct multi-granularity perception features for the description of moving objects. A two-level SVM classifier was used to classify pedestrians and vehicles in complex scenes.

Aiming at the problem of object classification under different angles and directions, Zhang et al. (2007) used the segmented local binary mode to describe the object area. After removing redundancy of object description features, the ECOC (Error Correcting Output Code) model was used to construct multiple classifiers, and the multiple classification problems were transformed into a combination of multiple dichotomizing problems to classify objects in common scenes. Fitzsimons and Dawson-Howe (2016) detected the left objects in the surveillance video, classified the four objects (messenger bag, trolley, people, crowd). It extracted the geometric features of the object area, such as area, perimeter, dispersion degree, external rectangle and SIFT features. Adaboost classifier, SVM and decision tree were used to cross-test the classification effect according to different features. Experimental results showed that the geometric feature was the best classifier and had strong robustness to classifiers and data sets. Bahman (2010) proposed an adaptive object classification method based on scene features to solve the object classification problem under different camera angles. It constructed a bilateral weight linear discriminant classifier by using the “screened” samples, the object classification effect of the classifier could be better improved. López et al. (2015) analyzed the moving object trajectory features in HOG-PCA feature space. KNN and dynamic HMM model were used to classify vehicles and pedestrians, it achieved better results. However, the above methods cannot extract the detail features, which will affect the final classification results.

Aiming at the problem that the shape and periodic motion features of moving objects in far-view monitoring scene could not be accurately recognized, a new multi-moving object classification algorithm was proposed in Alhadhrami et al. (2019). The algorithm adopted five eigenvalues (length-width ratio, area, velocity, position and orientation gradient histogram of frames), and used Bayesian classifier to realize the classification of people and cars in an intelligent community monitoring environment. In addition, in terms of the multi-feature fusion strategy, the weighted fusion method is generally adopted in the researches, and some researchers use fuzzy neural network and other methods to determine and update the weights. For example, Wu et al. (2018) conducted fuzzy modeling for features such as object area and shape complexity, proposed a corresponding fuzzy rule. It used a fuzzy neural network to optimize various parameters of the reasoning system to identify the object. Du et al. (2016) adopted the fuzzy integral technique for multi-feature fusion classification. In reference Bilik and Khomchuk (2012), aiming at the problem of tracking instability under complex background, an object tracking algorithm by fusing heterogeneous information with particle filter framework was proposed. Dong et al. (2021) proposed a multiscale feature extraction scheme based on spectral graph wavelet transform combined with improved random forest. The experimental results indicated that the proposed feature extraction method had high effectiveness and robustness. Elhawar et al. (2021) investigated the effect of different backbone feature extraction such as AlexNet, VGGNet, GoogleNet on an imbalanced small objects dataset after grouping them by shape and color in the Fully Convolutional Networks (FCN). Wan et al. (2021) introduced a new elastic feature extraction algorithm called the sparse fuzzy 2D discriminant local preserving projection. First, the membership matrix was calculated using the fuzzy K-nearest neighbors (FKNN), which was applied to the intra-class weighted matrix and the interclass weighted matrix. Second, two theorems were developed to directly solve the generalized eigen functions. Finally, the optimal sparse fuzzy 2D discriminant projection matrix was regressed using the elastic net regression. Liang et al. (2020) presented a novel convolutional auto encoders-based noise-robust unsupervised learning method for extracting high-level features accurately from aerial images and mitigating the effect of noise. The proposed method introduced the noise-robust module between the encoder and the decoder. Besides, several pooling layers in CAEs were replaced by convolutional layers with stride = 2. The proposed unsupervised noise-robust feature extraction method attained desirable classification accuracy in ideal input and enhanced the feature extraction capability from noisy input.

Although the scholars have done a lot of research on the object features extraction and achieved good results. Through comparison analysis, it is found that the commonly used object shape features are simple in calculation, they have good performance in some specific classification fields. However, when the object area detection is not accurate, the classification error is large, especially when the object area is disturbed by noise or video background, it is easy to reduce the detection accuracy of the object area.

In view of the above shortcomings, this paper proposes a multi-feature fusion strategy by combining aspect ratio shape feature, local binary mode feature, SIFT feature and semantic feature according to the characteristics of oil painting samples, and uses SVM classifier and KNN classifier to classify the oil painting images.

The arrangement of this paper is as follows: We present the modified faster RCNN in the modified faster RCNN. In the proposed multi-feature fusion strategy, the proposed multi-feature fusion strategy is introduced. Experiments and analysis gives the analysis of rich experiments. Conclusion gives a summary of this paper.

## Modified Faster RCNN

### ResNet-101 Network

**Figure 1:**
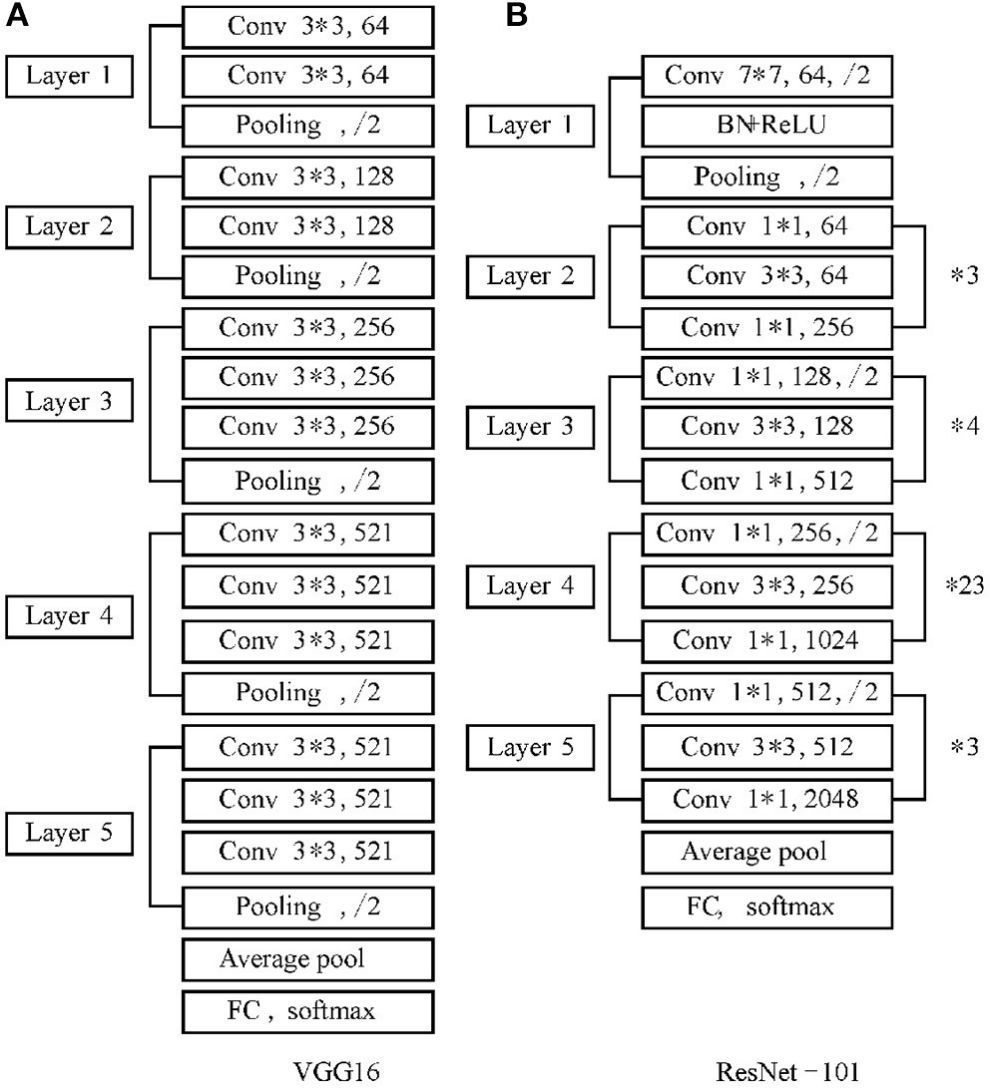
VGG-16 and ResNet-101 network structure.

The feature extraction Network is composed of Convolutional Neural Network (CNN). Wherein, the convolution layer, pooling layer, full connection layer and classification layer are the basic structures of CNN. The difference of CNN will affect the accuracy and efficiency of the object classification. The commonly used feature extraction networks are AlexNet, ZFNet, VGG-16, GoogleNet and ResNet. Both Googlenet and ResNet increase the depth of the network to optimize the model. However, the redundant network layer of GoogleNet learns parameters that are not identical mapping, this process results in the phenomenon that the accuracy of the training set decreases and the error rate increases. ResNet network solves the problem of model degradation mentioned above. A residual module is designed to allow the neural network to develop deeper and avoid the disappearance of network gradient, so that the model can achieve good learning effect. In order to improve the performance of feature extraction, ResNet-101 network is introduced to replace the original VGG-16 network as the feature extraction network to obtain a deeper fusion feature map. The network structure is shown in Figure 1B.

As can be seen from Figure 1, compared with the VGG-16 network, ResNet-101 adds a Batch Normalization (BN) layer following the convolution layer. The BN layer will normalize the input of each layer first and unify it into a normal distribution with a mean value of 0 and a variance of 1, which solves the problem that the data distribution of the middle layer changes in the training process of other networks. It also avoids the disappearance or explosion of the gradient and saves the training time.

Normalization equation (1) represents d-dimension input at each layer. Equation (2) represents the normalization of each dimension:

(1) $$\begin{array}{l} x=\left(x^{\left(1\right)}\cdots x^{\left(d\right)}\right) \end{array}$$

(2) $$\begin{array}{l} {x^{\prime }}^{\left(k\right)}=\frac{x^{\left(k\right)}-E\left[x^{\left(k\right)}\right]}{\sqrt{Var\left[x^{\left(k\right)}\right]}} \end{array}$$

Where *E*[*x*^(*k*)^] is the expect operation. *Var*[*x*^(*k*)^] is the variance operation. *x*^*i*^ denotes the pixel value.

### Improved RoI Pooling Layer

In the common two-stage test frameworks such as Fast-RCNN and Faster-RCNN, RoI pooling is used to pool the corresponding area into a uniform size feature map in the feature map according to the position coordinates of the candidate box for classification and regression. However, there is a two-time quantization process in RoI pooling operation. When the boundary coordinates of the candidate proposal box are quantized to integers and the initial regression position, a certain error will be generated. This error will directly affect the accuracy of detection, especially for small objects.

**Figure 2:**
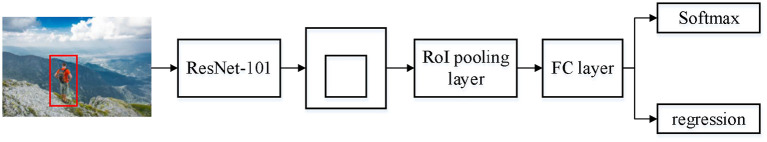
Improved RoI pooling layer.

Inspired by master R-CNN, this paper introduces RoI Align unit to address the above shortcomings in RoI pooling. ResNet-101 network is used to replace the original VGG-16 network as the feature extraction network. Figure 2 shows the RoI region pooling part of the improved faster R-CNN. When an image of 960 × 960 pixels is input, which contains a 315 × 315 pixels box to locate a person. After the image is processed by the ResNet-101 feature extraction network, the stride of the feature graph is 32. The RoI Align unit introduced in this article will retain floating point numbers, as shown by the arrow in Figure 2. The RoI Align unit eliminates the deviation of the border position caused by the quantization operation, and uses bilinear interpolation to obtain the image pixel values whose coordinates are floating point numbers. Thus it converts the whole feature aggregation process into a continuous operation, resulting in ideal detection accuracy.

### Soft-NMS Algorithm

Faster R-CNN algorithm adopts the traditional non-maximum suppression NMS algorithm for classification. The goal of this algorithm is to search for local maximum and suppress non-maximum elements. It can be expressed as equation (3).

(3) $$s_{i}=\{\begin{array}{ll} & s_{i},IoU(M,b_{i})<N_{t}\\ & 0,IoU(M,b_{i})\geq N_{t} \end{array}$$

Where, *b*_*i*_ is the i-th detection box. *s*_*i*_ is the score of the i-th detection box. *N*_*t*_ is the default threshold of NMS. M is the detection box with the highest detection score. *IoU*(*M, b*_*i*_) is the maximum crossover ratio of the i-th detection box *b*_*i*_ and the detection box M with the highest detection score.

The calculation formula of IoU in equation (4) is as follows:

(4) $$\begin{array}{l} IoU=\frac{A\cap B}{A\cup B} \end{array}$$

Where A is the area of the candidate box. B is the area of the original mark box.

It can be seen from equation (4) that the traditional NMS algorithm will set all frames adjacent to the detection box and greater than the preset threshold to zero. When detecting images with a high degree of overlap, the distance between the objects is very close. If the IoU value of the box with a low score and the box with a high score is greater than the preset threshold, the box with a low score will be directly suppressed, resulting in the failure of the object detection and thus affecting the detection performance of the model. In order to solve this problem, the Soft-NMS algorithm is introduced in this paper to replace the NMS algorithm, which is expressed as follows:

(5) $$s_{i}=\{\begin{array}{ll} s_{i}, & IoU(M,b_{i})<N_{t}\\ s_{i} & (1-IoU(M,b_{i})),IoU(M,b_{i})\geq N_{t} \end{array}$$

The Soft-NMS algorithm adopts the strategy of “weight penalty,” and an attenuation function is designed in the overlapping part of the adjacent detection boxes. Let it recursively re-score according to the current score instead of suppressing the adjacent boxes with low scores directly to keep the detection box of adjacent objects. The introduced super parameters in the Soft-NMS algorithm only appear in the test stage, and no super parameters are introduced in the training stage, which does not increase the computational complexity. According to the experiment results of Soft-NMS and NMS algorithms in reference (Yin et al., 2020) under different IoU thresholds, the value of IoU is set as 0.6 in this paper.

## Proposed Multi-Feature Fusion Strategy

**Figure 3:**
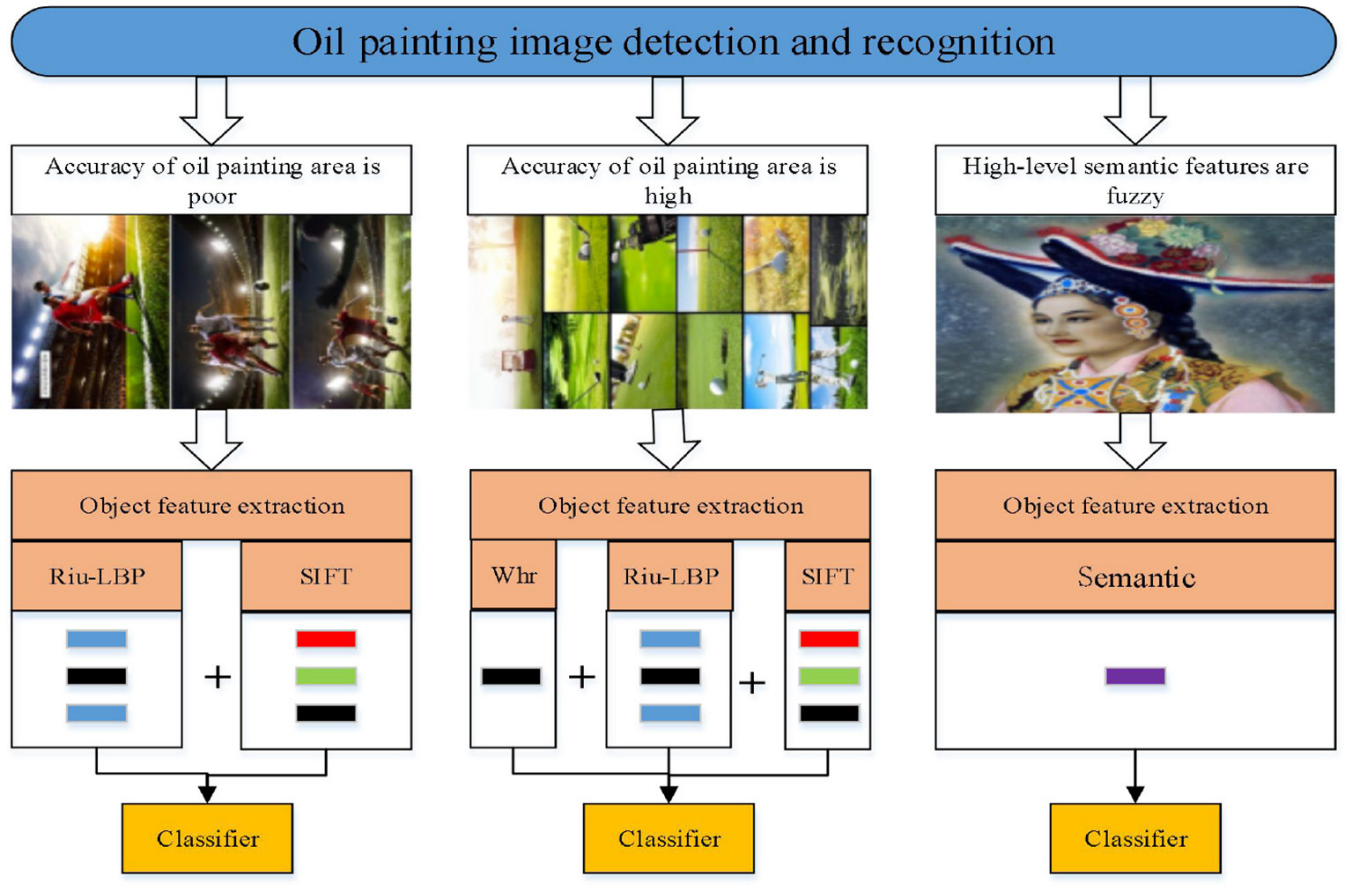
Feature extraction and classification for oil painting image.

Reasonable oil painting feature extraction can improve the accuracy of oil painting classification. In order to overcome the shortcoming of single feature extraction method, a feature extraction method combining multiple features is proposed, the process is shown in Figure 3. Firstly, the region of interest is extracted, and different feature extraction strategies are selected according to the different extraction accuracy of the oil painting area. The following features are mainly considered:

1. Whr (Width-height ratio).
  Whr feature refers to the width-height ratio of the minimum enclosing rectangle of the object. The Whr feature information of the object is constructed by obtaining the minimum enclosing rectangle of the object.
2. riu-LBP (rotation invariant uniform local binary mode).
  The rotation invariant uniform local binary mode is an improvement of the local binary mode (LBP). LBP feature is a non-parametric operator to describe the local spatial structure of an image. The basic idea is that the gray value of the central pixel is used as the threshold value, and the binary code obtained by comparing with the pixels in its neighborhood is used to express the local texture features. In order to solve the problem of rotation invariance of LBP operator, the riu-LBP operator is proposed. This feature has a strong robustness for light changes and scale changes and Angle changes of the object. While maintaining a strong descriptive ability and classification ability of the object, it greatly reduces the dimensional information of the object.
3. SIFT features.
  SIFT features have good invariance under the conditions of image rotation, scale transformation, affine transformation and perspective change. The features are consistent with the requirement of angle and variable scale, so it will be actively used in the feature extraction of oil painting image.
4. Semantic feature.
  Semantic feature can help scientists understand the high level information of image, which plays an important role in situational interpretation. The semantic feature is extracted by the improved faster R-CNN.

In this study, different feature extraction strategies are selected according to the different extraction accuracy of oil painting. As shown in Figure 3, when the extraction precision of oil painting area is not high, the riu-LBP and SIFT features are extracted. When the extraction precision of oil painting area is high, Whr, riu-LBP and SIFT features of the object area are extracted. Then, the bag-of-word model (He and Li, 2021) is used to transform SIFT feature matrix into feature vector to describe the oil painting. Meanwhile, the method of multi-feature fusion is used to describe the object. The object description feature vector is taken as the input of the next classifier. When the new sample is input, the classification sample is classified based on the trained classification model.

**Figure 4:**
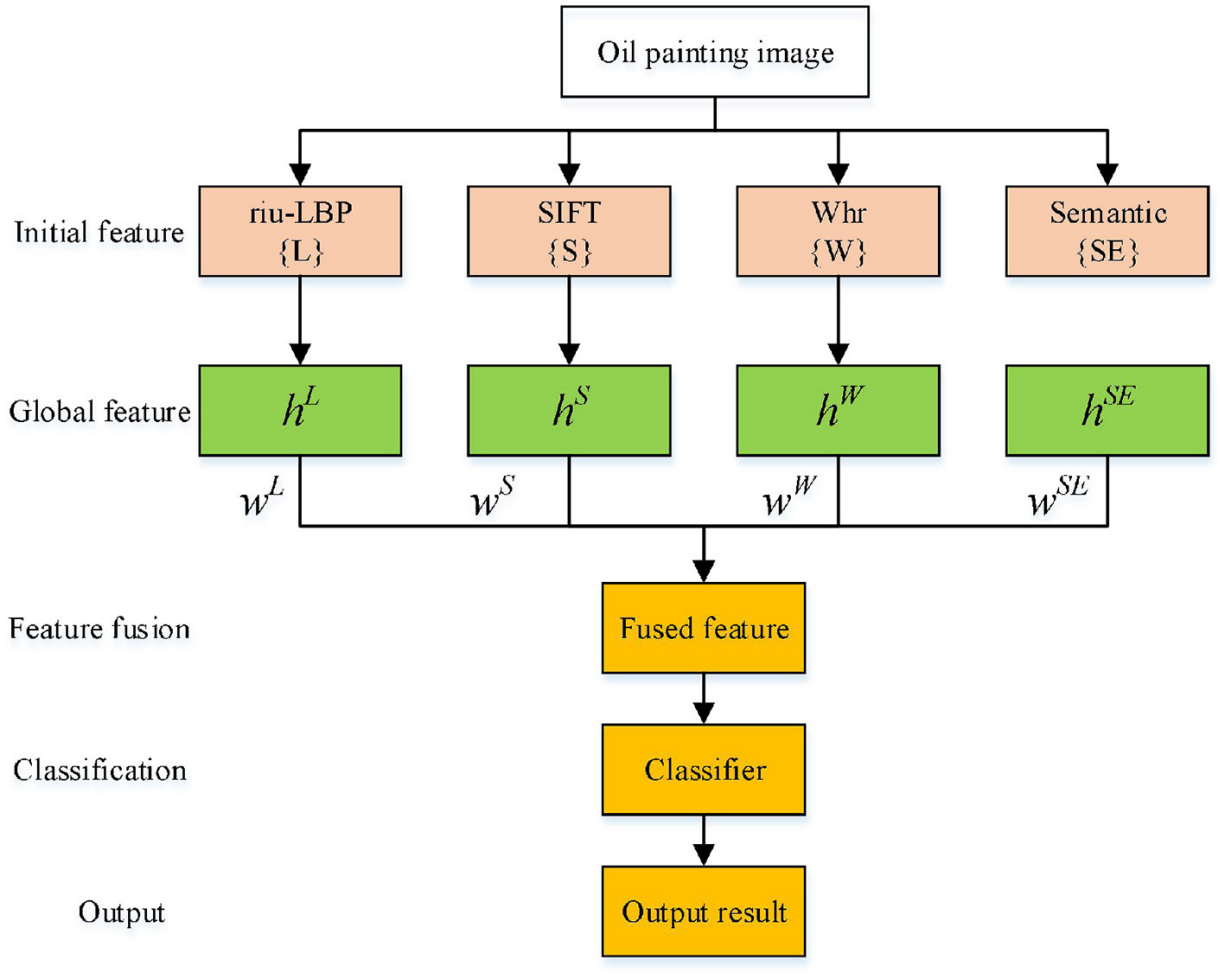
Multi-feature fusion process.

This paper mainly studies how to fuse multiple features to make the fused features have stronger descriptive ability for the object. The feature fusion framework is shown in Figure 4.

For the object classification problem, the weight sum of different feature vectors is directly considered, the weight coefficients are set to 1. Assuming that *h*^*L*^, *h*^*W*^, *h*^*S*^ and *h*^*SE*^ represent riu-LBP feature, Whr SIFT and semantic feature vector, respectively. The obtained vector V after weight sum is expressed as:

(6) $$\begin{array}{l} V=\left[h^{S}\;h^{L}\;h^{W}\;h^{SE}\right] \end{array}$$

## Experiments and Analysis

This experiment uses LibSVM and MATALB2017a as the experimental platform. First, the object training sample set and testing sample set are constructed.

**Figure 5:**
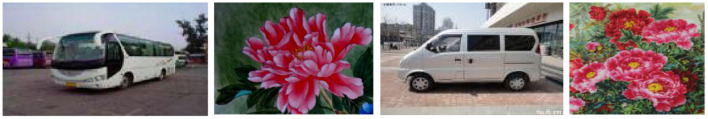
Sample A.

**Figure 6:**
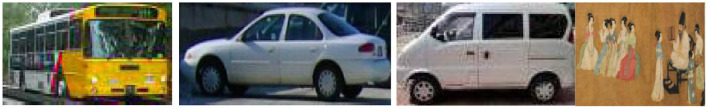
Sample B.

In the first experiment, multiple videos shot in the actual scene and the open video library of MIT University are adopted including pedestrian, car, bus and van. As shown in Figure 5, the extraction of moving object area in object sample set A is not accurate, the background removal is not complete, and the extracted object area is quite different from the object itself. The moving object area in object sample set B is extracted accurately. The moving object area of the sample is very close to the size of the moving object itself, and the background is removed thoroughly, which greatly reduces the influence of the background area on object classification as shown in Figure 6. A and B both contain 1,000 samples, each class has 250 samples. The sample is randomly divided into five equal sub-sample sets. Four sub-sample sets are selected for training, and the remaining is used for testing.

According to the idea of “one-to-one,” the SVM multi-classifier model is constructed, the features of the object region are extracted and used to train the SVM multi-classifier model. The training samples are used to construct the KNN multi-classifier model, and the last sample is used for classifier testing. The object description features and classifier models with the best classification effect are selected through experiments, then the classification effect is compared and evaluated.

In order to better evaluate the object feature extraction and the classification effect of the classifier, the classification accuracy is selected as the evaluation index. Assuming that there are *n* categories (*i* = 1,2,.,n), the classification accuracy of i-th class is defined as:

(7) $$\begin{array}{l} C_{i}=N_{i|i}/N_{i} \end{array}$$

Where *N*_*i*|*i*_ represents the originally sample number belonging to i-th class classified into i-th class. *N*_*i*_ is the total number of i-th class sample. For the i-th classifier, the higher classification accuracy denotes the better performance of the classifier. Here, the classification speed and other factors are not considered temporarily. According to the different features of the two sample sets, the riu-LBP texture feature and SIFT local feature of the object are extracted from the sample set A. Whr feature, riu-LBP texture feature and SIFT local feature are extracted from sample set B. The average classification accuracy of each feature is tested, and the experimental results are evaluated and analyzed. We first conduct experiments on the faster RCNN with fast RCNN and RCNN to show the effectiveness of faster RCNN, and apply it in the following experiments.

The Table 1 shows that Faster RCNN obtains the better results.

**Table 1:** Faster RCNN, fast RCNN and RCNN comparison/%.

**Method**	**Faster RCNN**	**Fast RCNN**	**RCNN**
Classification rate	89.6	81.7	76.2
Error rate	6.5	10.7	12.8

### Single Feature Extraction Experiment

For sample sets A and B, Whr, LBP, SIFT and semantic single features are used to conduct experiments, respectively. Since the extraction accuracy of oil painting in sample set A is not high, the Whr feature of the sample set A has no significance, so the Whr feature extraction experiment is not carried out. The experiment results of single feature extraction are shown in Table 2.

**Table 2:** Experiments on single feature extraction/%.

**Feature**	**SVM**	**SVM**	**SVM**	**KNN**	**KNN**
	**Line**	**RBF**	**(χ^2^)**	**eus**	**(γ^2^)**
**Sample set A**					
Whr	-	-	-	-	-
riu-LBP	42.5	41.6	52.2	72.3	73.6
SIFT	48.6	55.3	67.1	44.8	49.4
Semantic	51.2	60.7	71.5	55.7	53.4
**Sample set B**					
Whr	72.5	77.1	78.2	59.3	62.6
riu-LBP	47.6	51.5	62.7	69.6	75.4
SIFT	46.1	50.3	57.9	42.4	45.7
Semantic	52.8	59.6	62.7	57.3	55.4

According to the above experimental results, in the case of inaccurate extraction of object region of sample set A, the riu-LBP feature and KNN(γ^2^) classifier can achieve the highest average classification accuracy of 73.6%. For the accurate extraction of painting region in sample set B, the average classification accuracy of 78.2% can be achieved by using Whr feature and SVM() classifier. Because the oil painting area in sample set B is accurately extracted. Therefore, the shape features can greatly improve the object classification accuracy. Meanwhile, it can be seen from the experimental results that no matter what features are extracted, SVM classifiers using χ^2^-kernel function can achieve better classification effect than linear kernel function and RBF kernel function. KNN classifier uses χ^2^ distance to obtain better results than European distance. At the same time, it is found that the single feature extraction method of oil paintings has a low classification performance on the whole. Therefore, the object classification experiment based on the multiple features fusion strategy is further considered.

### Multiple Features Fusion Experiment

#### Experiments on Sample Set A

**Figure 7:**
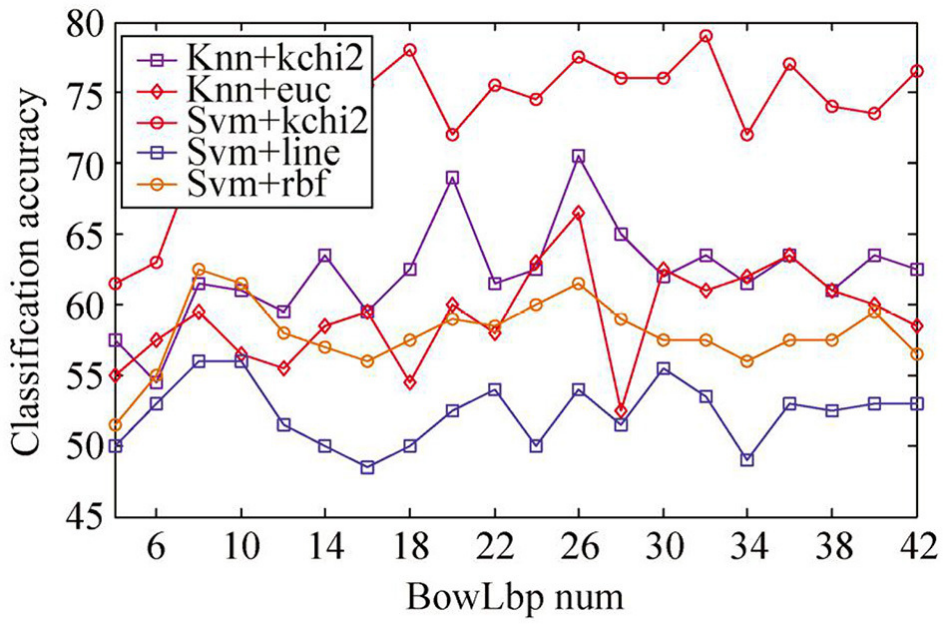
Classification accuracy of different classifiers with BowLbp feature on sample set A.

For sample set A, since the Whr feature in the sample set is not separable, only riu-LBP feature and SIFT feature are extracted. The BowLbp feature is obtained by fusing riu-LBP feature and SIFT feature. The classification accuracy of BowLbp features with different classifiers is shown in Figure 7. It can be seen from Figure 7 that the SVM(χ^2^) classifier is superior to other classification methods.

**Figure 8:**
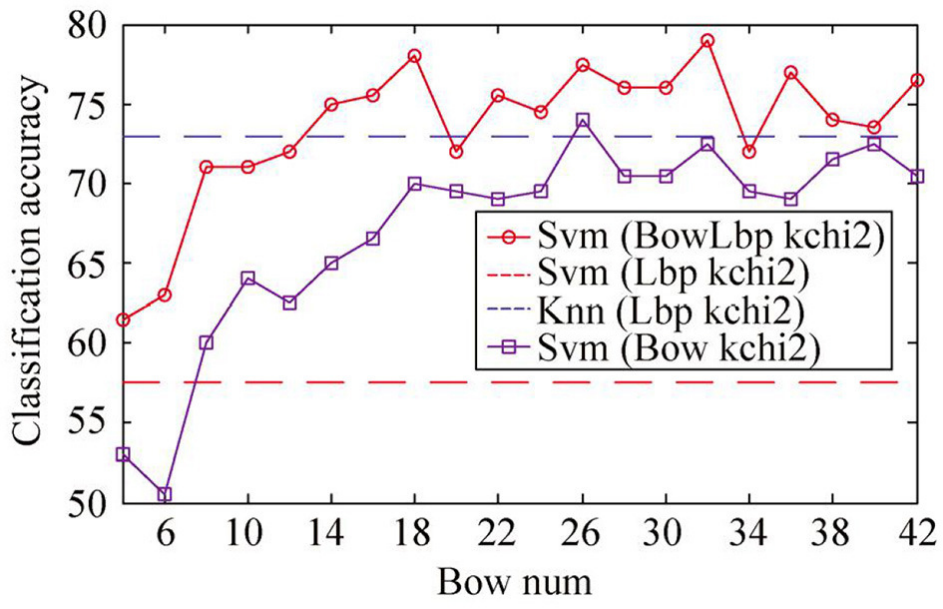
Comparison between BowLbp feature and single feature on sample set A.

The BowLbp feature is compared with the riu-LBP and SIFT. The classification accuracy of different object description features is compared as shown in Figure 8. It can be seen from Figure 8 that the BowLbp feature classification accuracy is better than riu-LBP feature and SIFT feature. Therefore, when the object area extraction accuracy is not high, the fusing Bow feature and riu-LBP feature can be used to classify the oil painting, which can greatly improve the average classification accuracy of the oil painting image.

#### Experiments on Sample Set B

Four oil painting description features including Whr feature, riu-LBP feature SIFT and semantic feature are extracted from the sample set B. There are five corresponding feature fusion methods. Different classifiers are selected for different feature fusion modes to conduct classification experiments, and the results are shown in Table 3.

**Table 3:** The classification result using different classifier on sample set B.

**Fused feature**	**SVM**	**SVM**	**SVM**	**KNN**	**KNN**
	**Line**	**RBF**	**χ^2^**	**euc**	**γ^2^**
BowLbpWhrSemantic	82.5	91.3	92.7	92.2	94.5
BowLbpWhr	78.2	83.4	85.5	82.8	84.8
BowLbp	63.1	67.6	75.1	58.0	63.1
BowWhr	76.3	80.4	80.7	78.8	80.1
LbpWhr	74.7	79.2	82.7	74.2	80.7

In Table 3, BowLbpWhrSemantic represents the fused features of SIFT, riu-Lbp, Whr, semantic. BowLbpwhr represents the fused features of SIFT, riu-Lbp, Whr. BowLbp represents the fused features of SIFT and riu-LBP. BowWhr represents the fused features of SIFT and Whr. Lbpwhr represents the fused features of riu-LBP and Whr. It can be seen from Table 3 that, for sample set B, the classification using fused features has better classification results than that of single feature. In the fused features, BowLbpWhrSemantic feature extraction algorithm can achieve better classification effect. And the SVM classifier based on kernel functions can achieve the best classification effect.

### Discussion

For sample set A, the classification method with the highest average classification accuracy in each feature is selected for comparison, and the results are shown in Table 4. Comparing the classification accuracy under different features, the BowLbp feature has the high classification accuracy.

**Table 4:** The classification result with SVM (χ^2^) on sample set A.

**Sample**	**Pedestrian**	**Car**	**Minibus**	**Bus**	**Color painting**
Lbp	59	72	35	75	81
SIFT	87	80	71	100	96
BowLbp	87	94	69	100	97

For sample set B, the classification method with the highest average classification accuracy in each feature is selected for comparison, and the results are shown in Table 5. Compared with the accuracy of each classification sample under different features, the BowLbpWhrSemantic feature has a higher classification accuracy for each classification. It maintains the advantage of single feature classification for the target. The experiment results show that the fused features have higher classification accuracy.

**Table 5:** The classification result with SVM (χ^2^) on sample set B.

**Sample**	**Pedestrian**	**Car**	**Minibus**	**Bus**	**Heavy color painting**
Whr	100	100	52	72	86
Lbp	74	89	47	74	89
SIFT	88	41	61	100	98
BowLbp	88	85	72	100	100
BowWhr	100	96	75	100	100
LbpWhr	100	100	58	85	99
BowLbpWhr	100	100	84	100	100
BowLbpWhrSemantic	100	100	100	100	100

For sports motion data set (Zhao and Chen, 2020; Li, 2021), the classification method with the highest average classification accuracy in each feature is selected for comparison, and the results are shown in Table 6. Compared with the accuracy of each classification sample under different features, the BowLbpWhr feature has a higher classification accuracy for each classification.

**Table 6:** The classification result with SVM (χ^2^) on sports motion data set.

**Sample**	**Leap**	**Shoot**	**Chuck**	**Hypsokinesis**	**Swimming**
Whr	65	72	45	58	64
Lbp	74	68	52	55	78
SIFT	58	71	47	62	77
BowLbp	81	85	63	73	95
BowWhr	85	87	75	73	96
LbpWhr	85	88	78	84	97
BowLbpWhr	96	95	97	100	100
BowLbpWhrSemantic	99	98	100	100	100

From the experiments on sports motion, the extracted fused feature can accurately describe the motion state and obtain better recognition effect.

## Conclusions

Oil painting feature extraction is the basis and key step of image classification. Use single feature extraction method cannot effectively detect the oil painting region due to the problems such as dimension, angle change etc,. Therefore, this paper puts forward a novel multi-feature fusion method for oil painting feature extraction and recognition based on SVM and KNN classifier. The new method combines shape features, local binary pattern, SIFT and semantic features of oil painting images. The experiment is carried out on three data sets including accuracy object area and inaccuracy object area. The experiment results show that the oil painting feature extraction combining multiple features can significantly improve the average classification accuracy and has better adaptability. Further research will focus on improving the classification accuracy, classification speed and other performances. More advanced deep learning methods will be utilized to extract richer features.

## Data Availability Statement

The original contributions presented in the study are included in the article/supplementary material, further inquiries can be directed to the corresponding author.

## Author Contributions

TC and JY: drafting and refining the manuscript. JY: critical reading of the manuscript. Both authors have read and approved the manuscript.

## Conflict of Interest

The authors declare that the research was conducted in the absence of any commercial or financial relationships that could be construed as a potential conflict of interest.
